# BetaBuddy: An automated end-to-end computer vision pipeline for analysis of calcium fluorescence dynamics in β-cells

**DOI:** 10.1371/journal.pone.0299549

**Published:** 2024-03-15

**Authors:** Anne M. Alsup, Kelli Fowlds, Michael Cho, Jacob M. Luber

**Affiliations:** 1 Department of Bioengineering, University of Texas at Arlington, Arlington, TX, United States of America; 2 Department of Computer Science and Engineering, University of Texas at Arlington, Arlington, TX, United States of America; 3 Multi-Interprofessional Center for Health Informatics, University of Texas at Arlington, Arlington, TX, United States of America; Albert Einstein College of Medicine, UNITED STATES

## Abstract

Insulin secretion from pancreatic β-cells is integral in maintaining the delicate equilibrium of blood glucose levels. Calcium is known to be a key regulator and triggers the release of insulin. This sub-cellular process can be monitored and tracked through live-cell imaging and subsequent cell segmentation, registration, tracking, and analysis of the calcium level in each cell. Current methods of analysis typically require the manual outlining of β-cells, involve multiple software packages, and necessitate multiple researchers—all of which tend to introduce biases. Utilizing deep learning algorithms, we have therefore created a pipeline to automatically segment and track thousands of cells, which greatly reduces the time required to gather and analyze a large number of sub-cellular images and improve accuracy. Tracking cells over a time-series image stack also allows researchers to isolate specific calcium spiking patterns and spatially identify those of interest, creating an efficient and user-friendly analysis tool. Using our automated pipeline, a previous dataset used to evaluate changes in calcium spiking activity in β-cells post-electric field stimulation was reanalyzed. Changes in spiking activity were found to be underestimated previously with manual segmentation. Moreover, the machine learning pipeline provides a powerful and rapid computational approach to examine, for example, how calcium signaling is regulated by intracellular interactions.

## Introduction

Insulin-secreting β-cells comprise the primary portion of groups of endocrine cells residing in the pancreas known as the Islets of Langerhans, making up approximately 50–70% [1–4]. Their role in the process of glycemic regulation has made them an ongoing source of study for Diabetes Mellitus. Type I Diabetes, specifically, is an autoimmune disorder characterized by the decreased secretion of insulin due to the destruction of β-cells. Complications from diabetes include micro- and macrovascular disorders such as thrombosis, atherosclerosis, and declines in cognitive function [5]. Two ongoing challenges to the treatment and/or cure of Type I Diabetes involve the reduced population of β-cells and the autoimmune attack within the body [6]. The main function of β-cells is the production and secretion of insulin. The release of this hormone is triggered by an increase in ATP [7] as the blood becomes oversaturated with glucose. As intense research efforts continue to explore this disease, many of the mechanisms behind insulin secretion are yet to be fully understood.

Insulin secretion is strongly correlated and regulated by calcium signaling. Glucose plays a large role in the stimulus of Ca^2+^ movement in the mitochondria, endoplasmic reticulum, nucleus, cytosol, and other areas of the cell and increases the level of cytosolic Ca^2+^ [8]. Additionally, an influx of Ca^2+^ through voltage-gated channels impacts insulin exocytosis and secretion. Insulin secretion patterns are typically studied by following the movement of Ca^2+^ oscillations (or spikes) in β-cells [9]. For example, membrane-permeable and high affinity calcium-sensitive fluorophores can be loaded into the cells. Upon binding free Ca^2+^ ion, fluorescent signals are generated and emitted when excited by an appropriate external light source. This process has been routinely captured in real-time subcellular imaging and allowed the investigator to track intracellular calcium levels. While the Ca^2+^ dynamics in individual cells has been monitored and tracked [10], challenges remain to examine the Ca^2+^ responses in each cell, especially in clusters of cells that are more physiologically representative of islets. Calculating control calcium activity provides a threshold that can be used to quantify significant changes in oscillations due to different forms of stimulation [11]. There is still much unknown regarding the effects of different stimuli on cell spiking activity, changes due to cell-cell interactions, and how insulin secretion fluctuates based on temporal or spatial variables. Moreover, measuring fluorescent intensities of individual cells that are clustered and/or overlap remains a daunting problem during image analysis. Efficient, accurate, and automated computational pipelines to determine potential cell-to-cell communication through Ca^2+^ oscillations are expected to enhance the current understanding of endocrine cell behaviors.

Automated image analysis tools are continually being developed and made readily available to investigators. Technological advancements have both decreased the time required to perform wet lab experiments and enabled researchers to obtain more sophisticated data. Open-source software such as ImageJ [12] and its updated architecture, Fiji [13], have been widely utilized for the evaluation of biological images. Fiji’s plugin, Trackmate [14], can further be used for the tracking of individual cells. Multiple other tracking procedures have been written in Python for specific research needs [15–17]. These tools are among many that can be combined with customized algorithms for enhanced biological research.

Segmentation is an important tool that allows researchers to computationally identify regions of interest (ROIs) and analyze them using different methodologies [12]. Manual segmentation has in the past been a standard protocol. However, it is time intensive and often subject to human errors and biases (S1 Fig) [18,19]. Typically, analysis of fluorescent images begins by outlining each specific cell to measure fluorescent intensities that might fluctuate with time. With the need for batch analysis of imaging data becoming more prevalent, an automated analysis tool is required to efficiently identify cells through a time-series image stack and eventually predict and validate physiological responses (e.g., insulin secretion). Hand segmentation remains a strong measure of evaluation for overall training accuracy, but automated methods are quickly being established as the new primary method of imaging analysis [20].

In recent years, many tools have been developed to continue improving automated segmentation of cellular microscopy images including watershed algorithms, pre-processing techniques, deep learning methods such as Convolutional Neural Network architectures, and combinations of multiple techniques [21–27]. Before the wide use of deep learning, novel segmentation techniques involved differentiating cells from the background through thresholding, masking general cell outlines through complex mathematical operations, redefining cell shapes with the watershed algorithm, incorporation of active contours to identify rounded cell shapes, or a combination of various methods to create a more accurate segmentation of clustered cells [28–31]. While these previous techniques are still applicable and useful in conjunction with newer methods, the incorporation of deep neural networks has been able to overcome common issues such as heterogenous distribution of the fluorescent markers for watershed and uniform shapes making masking difficult. Deep learning-based methods are able to utilize large hand annotated data sets to accurately segment cells without relying on predefined shapes, distinct cell boundaries, or an evenly distributed cell culture with minimal clustering [32–36]. 3D segmentation of sub-organelles within islets has allowed for more definitive quantification of intracellular interactions [37,38]. More novel deep learning networks, such as Cellpose, aim to create generalist models that increase flexibility of image analysis by training with a variety of cell types.

Cellpose [32] is a generalist, deep-learning algorithm designed for accurate segmentation of a wide variation of cell types. Its model is optimal for different types of biological research including 2D and 3D image sets, phase-contrast, and fluorescence microscopy as it provides segmentation without the need for retraining [32]. A variety of pre-trained models, named the ‘model zoo’, allows researchers to find the best segmentation styles for their image based on the large pre-segmented data sets (Cellpose, TissueNet, and LiveCell). The algorithm is user-friendly and customizable for specific experimental needs, and its newest updates offer human-in-the-loop pipelines and methods for retraining [39]. Since its inception, several studies have already utilized its model. Saad et al. [40] developed a procedure using Cellpose and Fiji to characterize ice crystals, and Hoeren et al. [41] adapted Cellpose into their pipeline analyzing cells adhering to PMP-ECMO membranes as an alternative segmentation process to Fiji. Reinbigler et al. [42] modified the model’s base framework and used it in conjunction with QuPath [43] to create a pipeline for the segmentation of Hematoxylin-Eosin (HE) stained histopathological whole slide images of myofibers. Waisman et al. [44] similarly used Cellpose for myofiber segmentation, outputting the images to ImageJ for ROI analysis. Fisch et al. [45] incorporated StarDist [33] for nuclear segmentation and Cellpose for nuclear segmentation in their automated workflow for the analysis of host-pathogen interactions. While Cellpose is a powerful tool, improvements are needed to obtain an end-to-end pipeline as segmentation is simply the first step of a long analysis process. BetaBuddy allows for complete analysis of image sets by automatically linking ROIs across frames, tracking fluorescence intensity, extracting cellular data (size, shape, intensity, etc.), and conducting initial statistical tests.

Our aim in this study was to develop and apply computational methods to obtain a faster, more accurate and in-depth analysis of calcium signaling in β-cells. We have developed a pipeline intended to decrease the time required for batch analysis of large image datasets and created a series of algorithms to automatically detect and calculate significant changes in fluorescent intensities.

## Materials and methods

**Fig 1:**
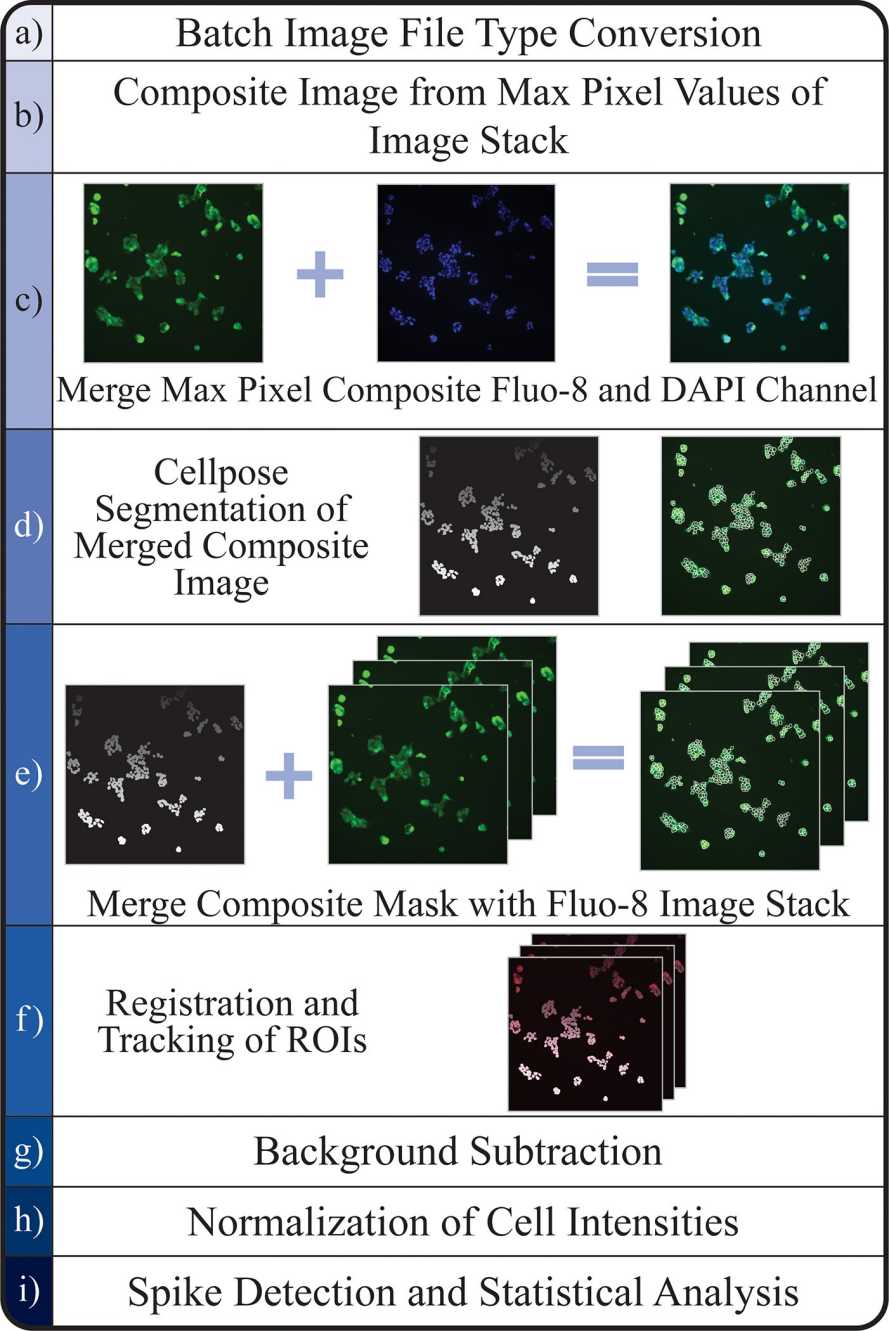
Pipeline flow chart. (a) Original Ca^2+^ microscopy images of β-cells (20x objective) converted to TIFF file from the Nikon-specific format. (b) A composite image is made by identifying the maximum value of each pixel location from the image stack. The resulting image is the brightest pixel out of all images in the stack, ensuring each cell is fully fluorescing in one image. This will allow for a more accurate segmentation without dropout due to non-fluorescing cells. (c) Composite Fluo-8 stained β-cell image and DAPI stain of nuclei of β-cells showing only the nuclei of each cell merged with both images in a separate channel, assisting in a more accurate segmentation. Detailed description of Cellpose is outlined in the next section. (d) ROI mask after composite segmentation and overlayed predicted outlines from after Cellpose segmentation. (e) Mask of segmented composite image is merged with all images in the image stack. Due to this step only image stacks with exceptionally minimal movement should be used. (f) Visualization of tracks created after registration and tracking. This figure includes the original fluorescent image, mask image, ROI outlines, and predicted tracks cells followed throughout the imaging process. (g-i) Statistical analysis is incorporated to generate automated results, which can be compared with manually analyzed results (see Fig 6 below).

Our primary goal is to create an automated system that outlines cell boundaries (segmentation) and track β-cells over a sequence of different time points (Fig 1). The system includes and performs statistical analyses on the calcium intensity patterns from individual cells. The completed pipeline can be used through a Jupyter Notebook and all package dependencies are described in the GitHub repository. Referred to as the BetaBuddy GitHub, it also contains installation and usage instructions for inexperienced programmers to easily follow and create a more accessible tool for all researchers to implement in their experimental procedures. The automated pipeline is expected to improve the accuracy of data and decrease the overall time necessary for future studies. The entire procedure was run using an NVIDIA DGX A100 High Memory GPU Supercomputer, containing 8 NVIDIA A100 high memory graphics cards with 80GB of GPU RAM each for a total of 640GB of GPU RAM for training large Deep Bayesian model architectures. The system also has 2 TB of HBM2 RAM and 128 AMD EPYC CPU Cores, and it connects to the internet via 10 GB/S connection. The process from image conversion, segmentation, tracking, and finally statistical analysis was successfully performed with 1 GPU, 12 CPUs, and 32GB of memory allocated.

### Calcium imaging and deep learning-based segmentation

The images used in our automated pipeline were sourced from previous experimental images that monitored and recorded Ca^2+^ dynamics in real-time [46]. Mouse derived βTC-6 insulinoma cells were cultured and allowed to grow for several days. A calcium indicator dye (Fluo-8) was used to measure changes in intracellular calcium levels. The cells were stained with 0.8 μM Fluo-8, and 1 drop/mL Nucblue to visualize the nuclei, for 30 min at 37°C and loaded onto a custom-designed electric field exposure chamber [46]. Non-invasive electric field stimulation (EFS; 15 min exposure) was chosen to manipulate the voltage-gated calcium channels and therefore modulate intracellular Ca^2+^ dynamics. A Nikon microscope was used to image the cells at 5 s intervals over a period of 2 min before and after EFS. This method provided a baseline and changes in the intracellular Ca^2+^ levels were measured. Data for such experiments consisted of stacked 2048x2048 pixel image sets of 25 frames before and after EFS. In total, 56 image stacks were analyzed. The Fluo-8 fluorescent image data sets consisted of pre- and post-exposure images of non-invasive 1 to 3 V/cm EFS. The analysis pipeline was used to analyze approximately 9–10 image sets for each experimental condition. Pre-exposure cells served as their own control.

Once the cells are imaged, multiple single or stacked images can be imported directly to the script and begin segmentation after format conversion. The Java library, Bio-Formats, is first used to convert the Nikon-specific file types to TIFF for ease of use in Linux ImageJ macro functions. The stack of images is then made into a single composite image with every pixel representing the maximum value of its pixel location. The final image will have the largest pixel value from all images in the stack. This was done due to the oscillating nature of Ca^2+^ signaling causing cells to be completely dark in certain frames. By creating an image with only the brightest pixels, we are able to ensure all cells able to uptake the fluorescence Ca^2+^ tag will be segmented and negate the issue of ROI drop-out caused by cells not fluorescing in all frames. Due to the clustering and static, non-migratory nature of β-cells, one mask image was determined sufficient in assisting segmentation for all frames. The composite image is then merged with an image of only the nuclei (labeled with DAPI) from the first frame of the exposure period (Fig 1C). It was determined that adding a DAPI channel greatly improved the segmentation process.

A validated deep learning-based generalist cellular segmentation algorithm, Cellpose, is used to automatically outline the boundary of each cell and create an ROI with user-defined parameters [32]. Users may specify if their images contain a DAPI channel, which channel contains the entire cell, the Cellpose model for segmentation, and thresholds. These parameters can be tested on one image to ensure quality segmentation. The generalist algorithm has a better performance with the addition of a DAPI channel due to the clustering nature of β-cells and difficulty differentiating neighboring cells with only one calcium indicator dye. After segmentation, an image defining the ROI of every cell is saved. The ‘mask’ image differentiates cells by assigning every ROI a different grayscale color (Fig 1D). The unique color can be used as a label during cell registration and tracking.

The mask image is replicated and merged with each frame of the original image stack without the DAPI channel in order to analyze the correct fluorescent intensity. Merging is performed by sorting the newly made mask image stack and original image stack into two different color channels (Fig 1E). This format will allow for the Fiji plugin, TrackMate, to begin cell registration by identifying the ROIs labeled in the channel containing the mask images [47]. The cell track detector analyzes the mask channel and registers every uniquely colored shape as a cell. Due to limitations of hand segmentation, including time of analysis and difficulty identifying cell boundaries across frames, 56–185 cells were identified in each trial. Manual segmentation is an established method of analysis in the field, and it is common practice to label a representative sample of cells that can be tracked over time for consistency (S1 Fig). As the automated pipeline is able to segment and register nearly every cell within the experimental population, 70–680 cells were labeled in each trial using BetaBuddy.

Next, each cell is linked across multiple frames, creating a “track” (Fig 1F). Lastly, fluorescent intensity for each frame in a cell’s track is obtained by referencing the first channel, which holds the original fluorescent image. The intensity values are taken from within the cell’s complementary ROI. This process is completely automated using ImageJ’s macro and python scripting features. This step will create an XML file that can be loaded into TrackMate and create a visualization of the tracks produced. Lastly, a CSV file is saved containing fluorescent intensity values, area, and spatial coordinate positions by grouping each cell into a “Track ID” over time.

### Background subtraction

**Fig 2:**
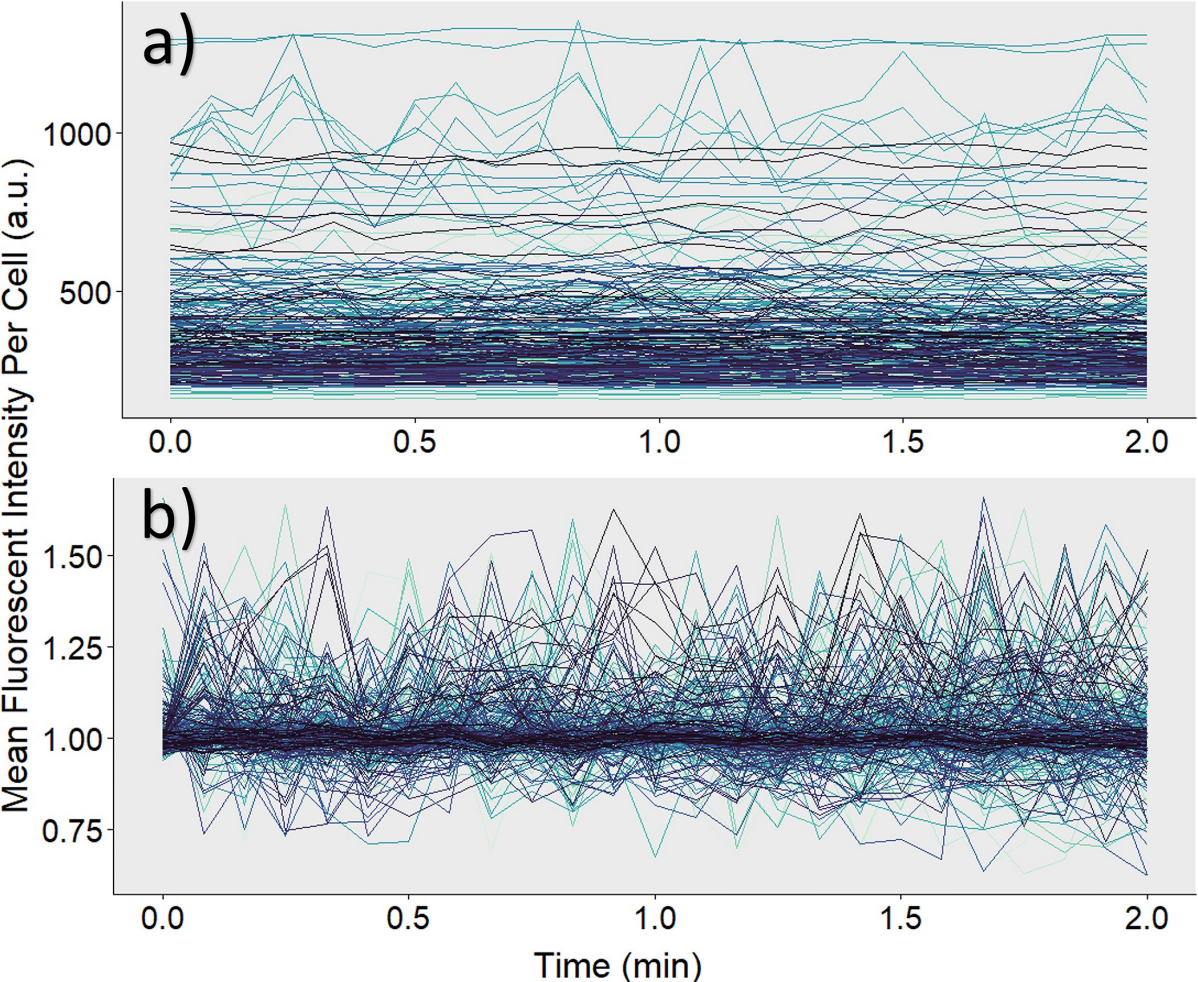
Intensity of experimental data. Each line represents an individual cell and its fluorescent intensity tracked over a period of 2 minutes. (a) Initial data is saturated with background noise. (b) Raw intensity values undergo background subtraction and normalization for a more accurate depiction of calcium activity.

Initially, the raw data CSV created after registration and tracking are very saturated with external background noise (Fig 2A). Fluorescent images typically have background noise associated with excessive dye or thermal electronic fluctuation. Therefore, raw data should undergo a background subtraction process. To obtain a well-distributed sample of background intensities, 100 pixel locations outside of the ROIs are randomly selected from the original image stack. The randomly selected pixel is compared to the mask image stack to ensure the pixel intensity has a value of 0, verifying it is not within an ROI.

Evaluation of all acquired data is analyzed through a procedure written in R and called upon through a terminal command in the Jupyter Notebook. The working directory is set to the user’s current operating space, and the CSV files containing the raw tracking information and background points are automatically imported. First, the average of all 100 randomly sampled background pixels is calculated for each unique frame. This mean value is then merged into the cell tracking information to create a singular data set. The average background intensity for each time point is then subtracted from the mean intensity of each cell within that frame. Next, to establish individual baseline activity, mean intensities were normalized specific to each individual cell.

### Normalization

Calcium intensities were normalized to account for different dye loading conditions that can be attributed to variations in cell size, cluster size, and potential differences in how each cell takes up the dye. This process allowed us to examine changes in fluorescent intensity in a way that could be fairly compared across all data points (Fig 2B). Normalization was performed by obtaining a “baseline” individual to each cell. In keeping with the original experiment’s methodology, a change in fluorescent intensity ≥10% was notated as spiking activity [46]. We kept this 10% threshold for the purpose of comparing the accuracy of either manual or automated data analysis of calcium dynamics in β-cells. To find a baseline for each cell, the number of data points until each specific Track ID reached a change of ≥10% from its first recorded intensity was calculated, and the mean of these first *n* points was divided from each background-subtracted intensity within the group. If a cell never reached an intensity level greater than 10% of its first value, then its baseline was determined using the mean intensity of all recorded data points for that cell.

### Spike determination

**Fig 3:**
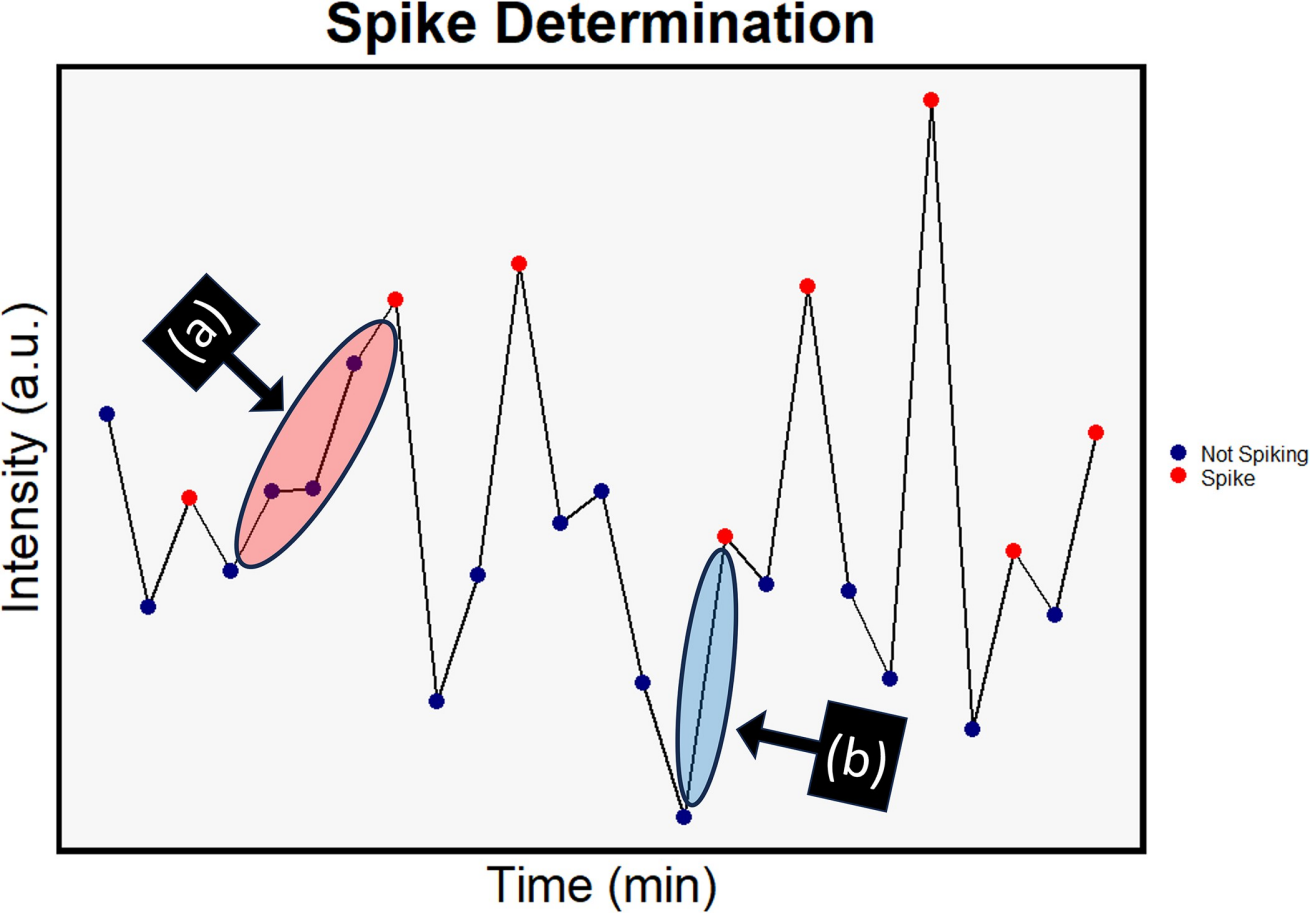
Calcium spike determination. Computational methods were used to automatically designate calcium spikes at a 10% threshold. This threshold was met through either (a) the sum of percent changes between two designated peaks or (b) a singular percent change of ≥ 10% immediately preceding a defined peak. The line graph represents fluctuations in fluorescent intensity over time for a single cell in a trial.

The purpose of this experiment was to repeat the analysis of spiking activity within the original data set that utilized hand segmentation methods. Our goal was to determine if there was any significant loss of data in the original methodology. For the automated analysis, we defined a calcium spike using the 10% threshold, as previously stated. The percent change in normalized intensity from its previous value was calculated for each βTC-6 cell. Based on the value changes, a spike was defined in two ways: either (a) the sum of the cumulative percent changes between two peaks was ≥ 10% or (b) the percent change from the data point immediately preceding any peak in intensity was ≥ 10% (Fig 3).

Using these parameters, we quantified each instance individual cells spiked over the course of the imaging period before and after external stimulation. Mean activity for each trial was calculated from the aggregate of all spikes within the population.

## Results

### Statistical analysis

Prior to statistical tests, outliers for singular trials were detected using a standard Z score. Track IDs with any intensity values greater than a Z score of 3.0 or less than -3.0 were automatically removed. Mean spiking activity was calculated as the total number of spikes recorded for each trial divided by total number of cells over time.

**Fig 4:**
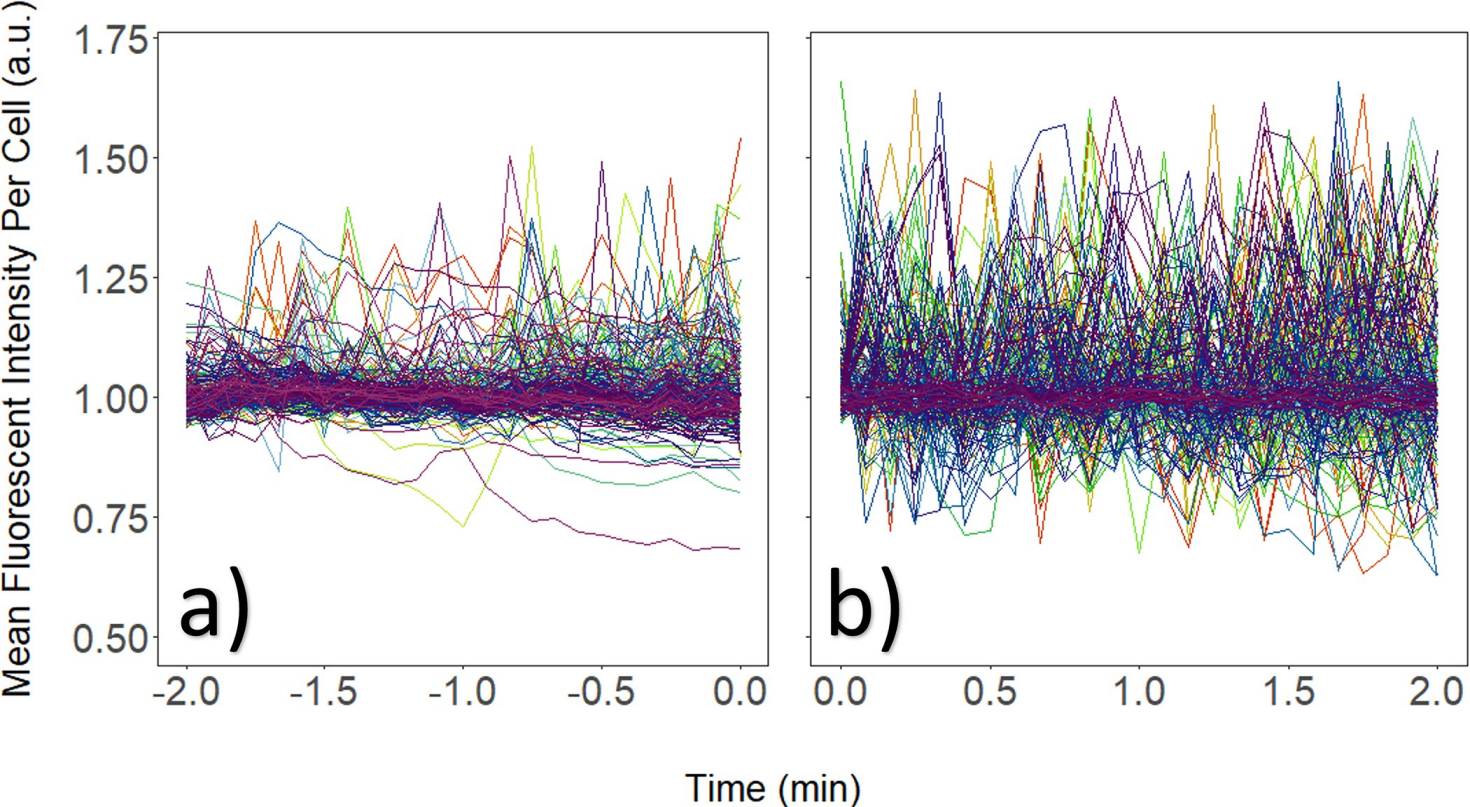
Signaling changes post EFS stimulation. Variations in activity, including changes in the fluorescent intensity and spiking frequency, increased significantly following a 15 min exposure to 1 V/cm EFS. (a) Fluorescent activity prior to stimulation. (b) Fluorescent activity post EFS.

Fig 4 illustrates one experiment using 1 V/cm stimulation. Normalized calcium activity was plotted from -2 to 0 min (i.e., control before EFS was applied) and from 0 to 2 min following 15 min EFS. Calcium recording during 15 min EFS was not performed to minimize fluorophore degradation and/or bleaching.

**Fig 5:**
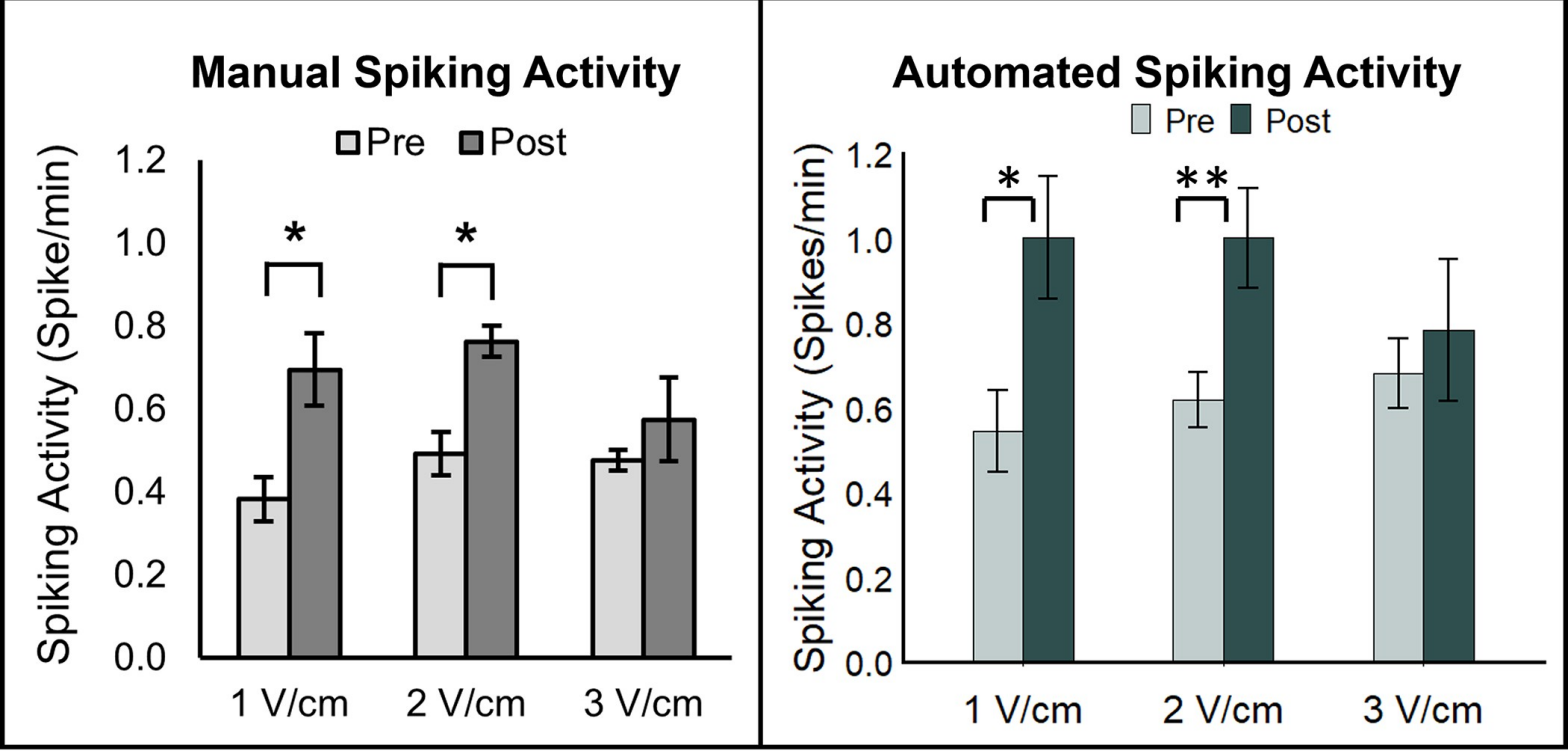
Comparison of results. The number of calcium spikes determined by hand are shown in the left panel. Using the same images, the results from the automated analysis are shown in the right panel. * denotes p < 0.05 and ** p < 0.01 using a two-tailed paired t-test.

A two-tailed paired *t* test was performed to evaluate significant changes in the calcium signaling of the β-cells in response to EFS of 1 to 3 V/cm. The numbers of spikes calculated through the automated pipeline were plotted and compared with published results (Fig 5).

**Fig 6:**
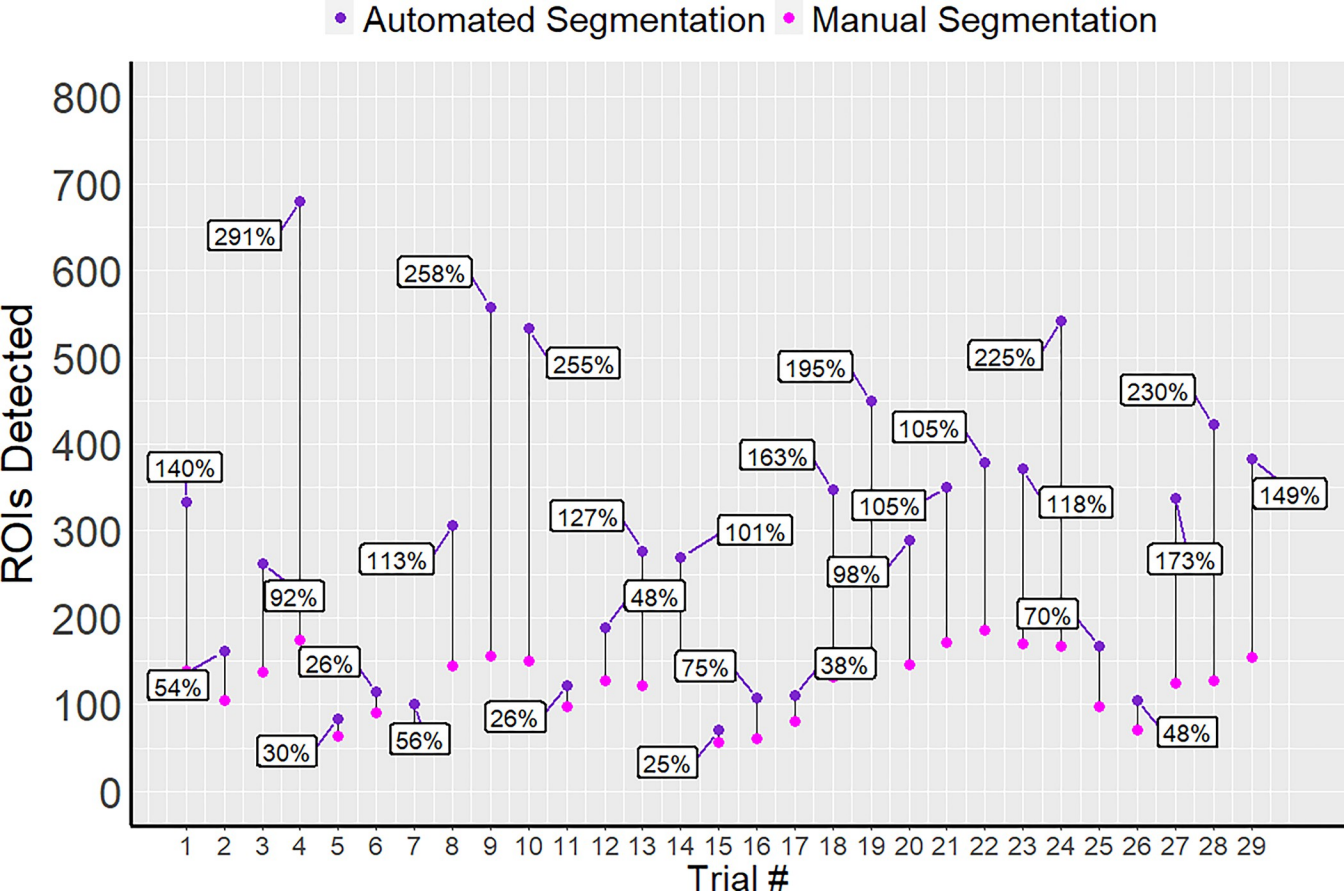
Manual vs Automated ROI identification. The total number of ROIs identified using manual and automated segmentation can be compared. The percent change between the two methods is displayed for each trial. The difference in labeled ROIs between the two segmentation methods ranged from 25–291% per trial.

Results obtained through automated methods validated the findings previously recorded using manual analysis. EFS of 1 and 2 V/cm significantly increased the calcium spiking activity compared to their respective controls. There was no significant increase in calcium activity found at 3 V/cm. In further comparison of the manual vs. automated analysis, it was noted that the number of calcium spikes calculated using hand segmentation were underestimated by ~40% in all control and experimental conditions with respect to their counterparts in the automated results. This trend is indicative of systematic errors that may have been caused by human biases. For example, one factor that could account for the discrepancy in these calculations could be attributed to a limited number of ROIs in the manual segmentation (Fig 6).

ROI identification is often constrained with manual segmentation practices. Images may only have active cells and/or cells with clear boundaries registered due to visual impairment and time constraints. Which ROIs are identified can also be a source of bias. Thus, calculations of spiking activity may only be sourced from a limited number of cells. In contrast, automated segmentation using BetaBuddy resulted in near complete registration of all cells present within each frame.

## Discussion

The application of electric field stimulation to βTC-6 cells was found to successfully increase calcium spiking activity. This indicates that EFS within 1 to 2 V/cm serves as a potentially beneficial treatment for pre-conditioning β-cells for enhanced insulin secretion. It is interesting to note that a larger EFS (3 V/cm) did not induce statistically significant changes in the calcium spiking determined either by hand or by automated pipeline. We speculate that, since the induced electrical potential depends on the cluster size, larger EFS causes non-negligible and perhaps irreversible changes in the cell membrane potential. Generally, automated analysis found greater overall spiking activity in the βTC-6 cells. While it remains to be further elucidated whether one-time treatment with EFS is sufficient to pre-condition pancreatic cells, it does offer a non-biologic approach to regulate insulin secretion and/or trafficking.

**Fig 7:**
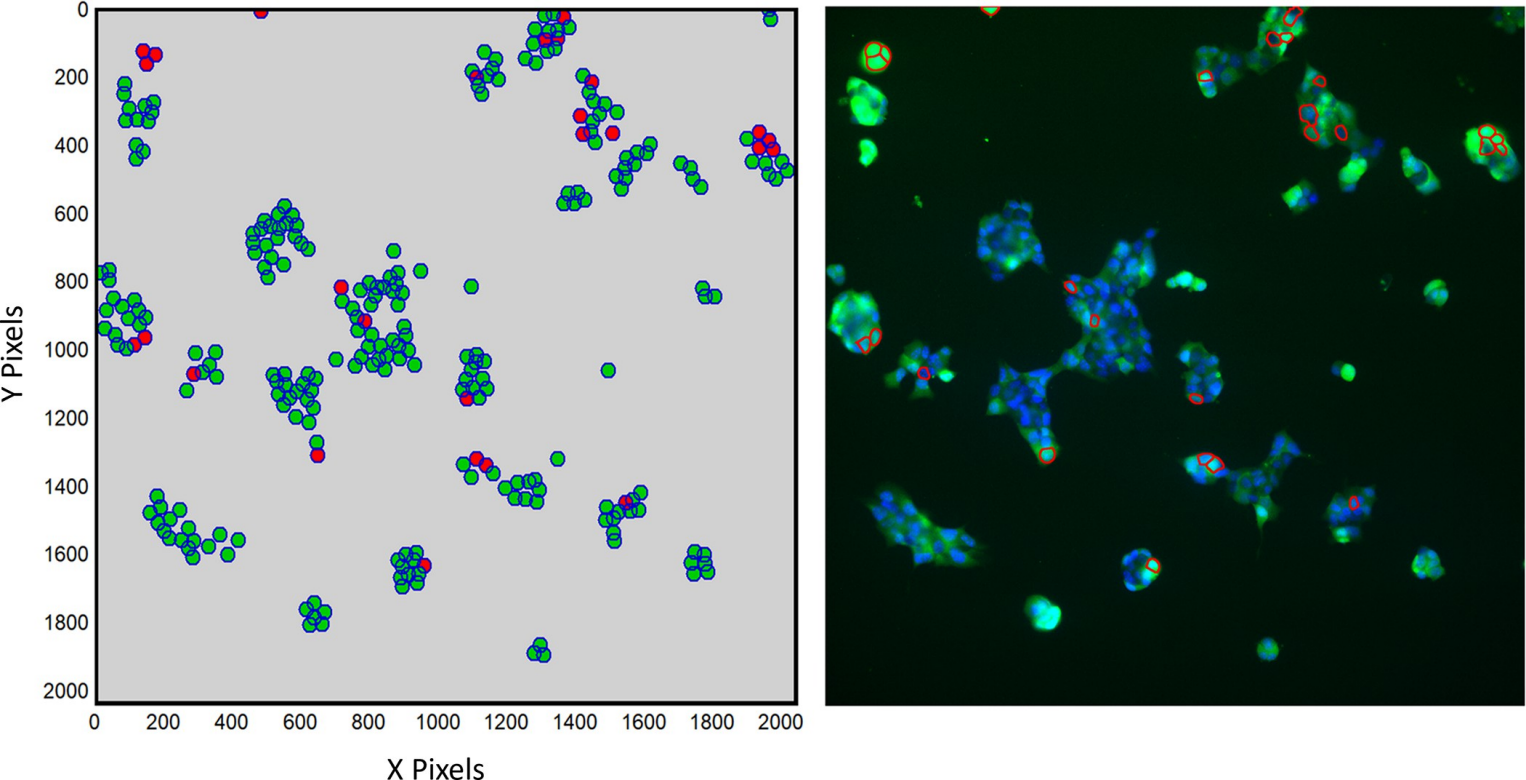
Spatial visualization. a) Cartesian coordinates were used to visualize the locations of spiking cells. Only cells currently spiking are outlined at a given time. The cells highlighted in red color, for example, exhibit calcium spiking activity at t = 25 s. The visual analysis does not correlate to the actual size of cell clusters. b) The generated spatial data can be used to trace back and identify cells of interest in the original experimental images.

Furthermore, results that were previously reported using manual segmentation (see Fig 5) were compared with those determined by BetaBuddy. Both sets of results were comparable, suggesting analysis by hand was able to quantify the calcium dynamics in β-cells but underestimated the calcium spiking rate. Automated segmentation thus appears to be a viable and more accurate method for obtaining reliable results without the tedious and laborious practices. Development of the BetaBuddy pipeline therefore enables us to predict the optimal application of exogenous stimuli that can be validated experimentally and provides a pathway to fully utilize the machine learning-based discoveries in the β-cell physiology. One novel method of data processing includes isolating active cells from the general population. Quantification of spikes from individual cells provides specialized data that allows us to track the active population between the control and experimental sets (S2 Fig). Active cells can be defined as any cell that spikes at least once during an imaging period (see Fig 7). These groups can be compared prior to and following external stimuli to determine any notable changes in calcium activity.

There are many methods through which insulin and Ca^2+^ activity can be stimulated. Pharmacological stimulation has been shown to increase calcium activity through drugs such as tolbutamide, which closes the K_ATP_ channels of the β-cells [48,49]. In addition to EFS, non-invasive stimulation can occur through applications such as photobiomodulation (PBM) therapy [7]. Using this pipeline, the direct comparison of changes in calcium dynamics between different stimuli can now readily be explored.

### Future work

We plan to continue expanding the accessibility of our pipeline. A cleaner, more interactive user interface within the Jupyter Notebook is being developed along with an Anaconda package for researchers to easily create an environment with all the necessary packages pre-installed. Additionally, we are developing more user-friendly options for running different data analyses. This includes options for various statistical tests depending on researcher preference and the types of biological questions being asked. With these new features, users will be able to run desired tests within the pipeline along with having access to both the raw and normalized data.

We intend to use this improved process to first reanalyze a previously hand-segmented data set exploring the effects of PBM therapy on the Ca^2+^ oscillations in β- and α-cells [7]. PBM is believed to increase insulin and glucagon secretion through accelerated ATP synthesis caused absorption of near-infrared light by the cytochrome c oxidase enzyme in the mitochondria [50–52]. We will also be developing β/α-cell co-cultures to explore how calcium activity is affected through intercellular modulation and determine any changes in signaling responses post-PBM.

In future studies, spatially resolved interpretation of data offers an alternative to traditional statistical methods of analyzing calcium signals. Cell-specific spatial and fluorescent intensity data can be utilized, for example, to evaluate varying levels of insulin secretion in given locations (Fig 6). For example, β-cell location and population within an islet has been shown to strongly impact the overall islet dynamics [53]. Researchers can use these methods to not only look at current spiking patterns, but also begin to analyze how calcium signaling is affected by intercellular interactions.

Questions regarding calcium spiking frequency and cell-to-cell interaction through calcium propagation are now feasible for detailed analysis. This is perhaps one of the major advantages that were made possible by the development of automated segmentation and data evaluation. For example, intercellular communication often relies on calcium waves through gap junctions [54–56]. Since EFS we used in this work does not penetrate the cell, it may be hypothesized that the outer cells in a cluster of cells are likely affected by the EFS and generate calcium activities first. Through calcium intercellular communication, the inner cells in the cluster are affected at a later time. However, the cluster is collectively activated by EFS that leads to an increase in insulin production. Experiments are underway to validate this hypothesis and the results will again be used to train the model to recognize the calcium-dependent cell-to-cell communication that may be relevant to the islet physiology.

## Conclusions

Our current pipeline successfully isolates β-cells, tracks calcium signaling patterns over time, and produces preliminary data values that can be used in future statistical analyses. Automated segmentation reduces biases and decreases time required for analysis compared with manual segmentation. With this information, we are able to pinpoint individual cells with the most biologically significant spiking patterns. Additionally, individual calcium activities can be monitored over time, offering critical spatial and temporal information.

BetaBuddy has a multitude of benefits for potential research. Its customizable platform allows it to be adapted for use with multiple cell types. Its base structure has already been utilized for the automated analysis of mouse brain endothelial cells (MBECs) and human mesenchymal stem cells (hMSCs). Furthermore, the pipeline is designed in a way that researchers from diverse backgrounds can use it regardless of past coding experience.

This first analysis serves as a reliable test case for BetaBuddy’s ability to batch analyze data. From here we will seek to apply the developed pipeline to evaluating other forms of stimuli (e.g. chemical/physical). Information obtained from this data may greatly assist in treatments for Type I Diabetes. The first step to developing a more quantifiable method for determining viable cells is obtaining a better understanding of the calcium dynamics of both β- and α-cells. Co-culturing these cell types together will provide insight into how different forms of stimulation might improve cell viability in an in vivo setting. Moreover, our automated pipeline can decrease evaluation times for multiple treatment alternatives and even serve as a drug repurposing tool.

## Supporting information

S1 Fig Manual vs. Automated segmentation. Deep learning algorithms have been found to greatly improve segmentation accuracy. (a) Previous hand segmentation methods often undersegmented the total ROIs present, often ignoring highly clustered areas. (b) Our automated system comprising merging DAPI with the targeted fluorescein channel, segmentation, and subsequent ROI tracking was able to consistently identify more cells at a higher accuracy and track localized signals.
(TIF)

S2 Fig Effects of EFS on active cell population. Each point represents the number of active cells within a specific trial. Active cells were defined as any cell that spiked at least once during the trial’s imaging period. The change in population following EFS stimulation can be tracked across each connecting line. The mean, upper quartile, and lower quartile of the active populations pre- and post-EFS are represented with their respective boxplots.
(TIFF)
