# Supplementary figures and images for: BetaBuddy: An automated end-to-end computer vision pipeline for analysis of calcium fluorescence dynamics in β-cells
Source: PLoS One. 2024 Mar 15;19(3):e0299549. doi: 10.1371/journal.pone.0299549 (PMC10942061; doi:10.1371/journal.pone.0299549)

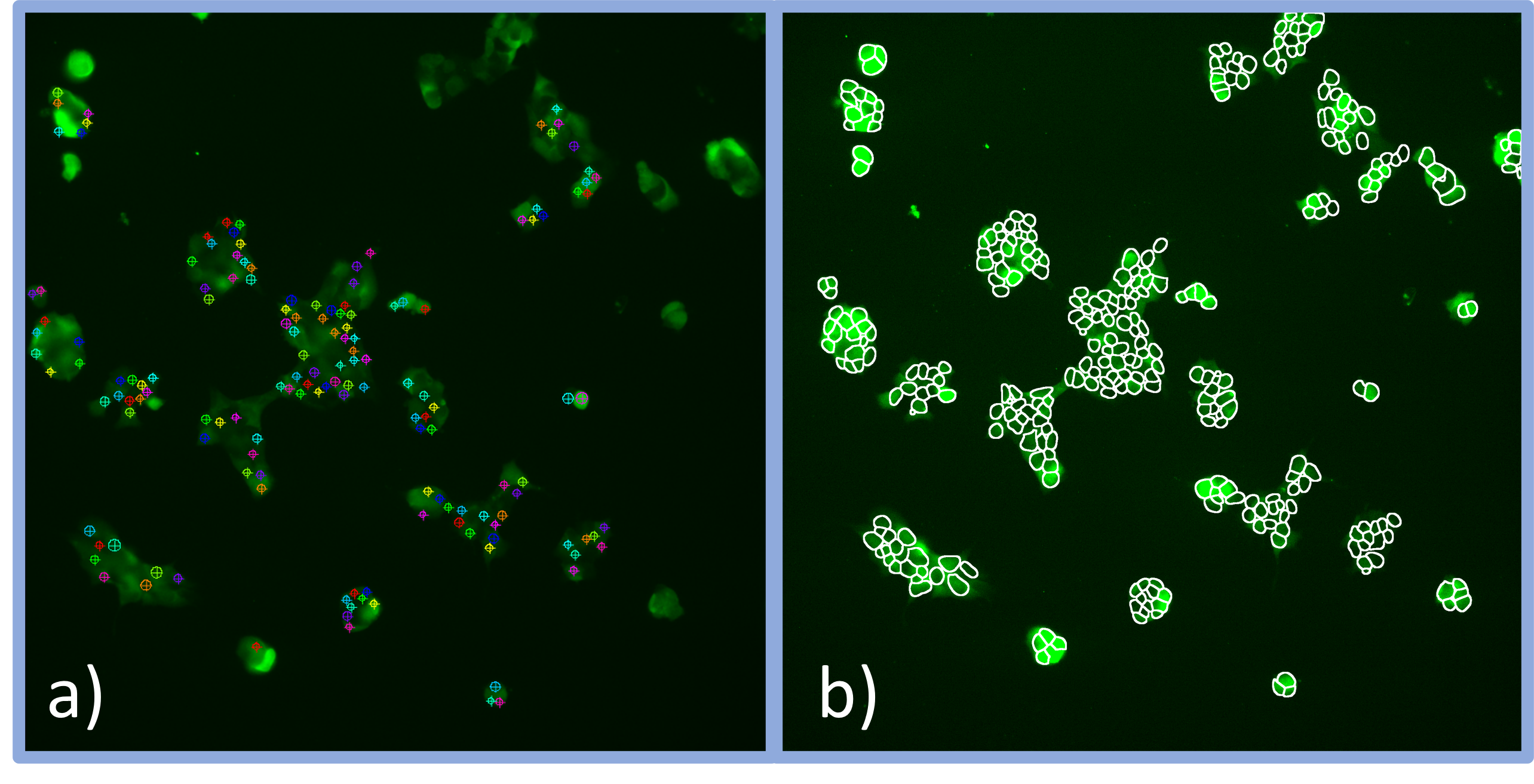

Supplement: S1 Fig — Deep learning algorithms have been found to greatly improve segmentation accuracy. (a) Previous hand segmentation methods often undersegmented the total ROIs present, often ignoring highly clustered areas. (b) Our automated system comprising merging DAPI with the targeted fluorescein channel, segmentation, and subsequent ROI tracking was able to consistently identify more cells at a higher accuracy and track localized signals. (TIF) [file pone.0299549.s001.tif]

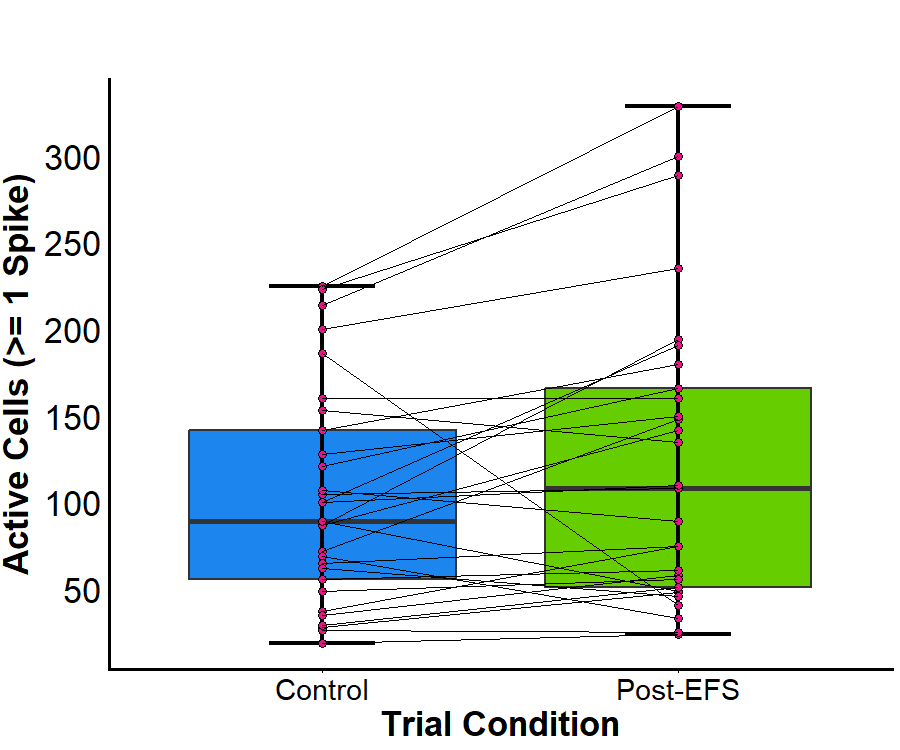

Supplement: S2 Fig — Each point represents the number of active cells within a specific trial. Active cells were defined as any cell that spiked at least once during the trial’s imaging period. The change in population following EFS stimulation can be tracked across each connecting line. The mean, upper quartile, and lower quartile of the active populations pre- and post-EFS are represented with their respective boxplots. (TIFF) [file pone.0299549.s002.tiff]
